# Genome-Wide Association Study for Biomass Related Traits in a Panel of *Sorghum bicolor* and *S. bicolor* × *S. halepense* Populations

**DOI:** 10.3389/fpls.2020.551305

**Published:** 2020-11-12

**Authors:** Ephrem Habyarimana, Paolo De Franceschi, Sezai Ercisli, Faheem Shehzad Baloch, Michela Dall’Agata

**Affiliations:** ^1^CREA Research Center for Cereal and Industrial Crops, Bologna, Italy; ^2^Department of Horticulture, Faculty of Agriculture, Ataturk University, Erzurum, Turkey; ^3^Faculty of Agricultural Sciences and Technologies, Sivas University of Science and Technology, Sivas, Turkey

**Keywords:** *Sorghum bicolor*, *Sorghum halepense*, biomass, SNP markers, marker haplotypes, GWAS

## Abstract

The efficient use of sorghum as a renewable energy source requires high biomass yields and reduced agricultural inputs. Hybridization of *Sorghum bicolor* with wild *Sorghum halepense* can help meet both requirements, generating high-yielding and environment friendly perennial sorghum cultivars. Selection efficiency, however, needs to be improved to exploit the genetic potential of the derived recombinant lines and remove weedy and other wild traits. In this work, we present the results from a Genome-Wide Association Study conducted on a diversity panel made up of *S. bicolor* and an advanced population derived from *S. bicolor* × *S. halepense* multi-parent crosses. The objective was to identify genetic loci controlling biomass yield and biomass-relevant traits for breeding purposes. Plants were phenotyped during four consecutive years for dry biomass yield, dry mass fraction of fresh material, plant height and plant maturity. A genotyping-by-sequencing approach was implemented to obtain 92,383 high quality SNP markers used in this work. Significant marker-trait associations were uncovered across eight of the ten sorghum chromosomes, with two main hotspots near the end of chromosomes 7 and 9, in proximity of dwarfing genes *Dw1* and *Dw3*. No significant marker was found on chromosomes 2 and 4. A large number of significant marker loci associated with biomass yield and biomass-relevant traits showed minor effects on respective plant characteristics, with the exception of seven loci on chromosomes 3, 8, and 9 that explained 5.2–7.8% of phenotypic variability in dry mass yield, dry mass fraction of fresh material, and maturity, and a major effect (*R*^2^ = 16.2%) locus on chromosome 1 for dry mass fraction of fresh material which co-localized with a zinc-finger homeodomain protein possibly involved in the expression of the *D* (Dry stalk) locus. These markers and marker haplotypes identified in this work are expected to boost marker-assisted selection in sorghum breeding.

## Introduction

Sorghum [*Sorghum bicolor* (L.) Moench] is the fifth cereal in the world in terms of production and acreage (Ordonio et al., 2016a). The extent of sorghum cultivation, its resilience to biotic and abiotic stresses, adaptability to diverse environments, low agricultural inputs requirements, and its use as functional food with good nutritional value and high content in health-promoting compounds make it an important staple crop to enhance food security across the globe (Awika and Rooney, 2004; Dykes, 2019; Przybylska-Balcerek et al., 2019). Besides its use for human and animal nutrition, the increasing demand for sustainable and renewable energy sources stimulated the cultivation of sorghum as an energy crop. Sorghum has drawn the interest of the scientific community as a model for the study of bioenergy crops thanks to its optimal features, including high biomass yields, quick growth, C4 photosynthesis pathway, stress tolerance and, not least, its small genome size (Mullet et al., 2014; Sadia et al., 2018). The *S. bicolor* genome sequence was first released in 2009 (Paterson et al., 2009) and the current version is 3.1.1; it is sized 732.2 Megabases (Mb), arranged in *x* = 10 (2*n* = 20) chromosomes and reporting more than 34,000 annotated genes several of which can be harnessed in genetic introgressions to improve biomass production in this crop (McCormick et al., 2018).

Available scientific evidence showed that sorghum genetic improvement can greatly benefit from the genomes of wild relatives (Habyarimana et al., 2018). Johnsongrass [*Sorghum halepense* (L.) Pers.] is one of the wild species of interest; it is a natural allotetraploid (2*n* = 40) thought to have originated by the spontaneous hybridization between diploids *S. bicolor* and *S. propinquum* (Kunth) Hitchc., followed by chromosome doubling (Paterson, 2008). Its highly efficient system of reproduction and propagation makes it one of the world’s most aggressive grass weeds (Kaur and Soodan, 2017). However, the interest toward this species rose among sorghum breeders due to its ability to transmit a strong perenniality to the progeny from hybridizations with domesticated *S. bicolor* (Cox et al., 2002, 2010, 2018b; Piper and Kulakow, 2007; Habyarimana et al., 2018). Perennial crops are considered a paradigm shift in modern farming owing to their potential to help the world move toward more sustainable production and environment friendlier systems to increase food security while reducing tillage, water consumption, soil erosion and CO_2_ emissions (FAO, 2013). Cultivated sorghum could therefore benefit from perenniality not only in terms of food, fodder and energy security, but also in terms of sustainability, cutting environmental load through increased energy balance, and soil protection (Hallam et al., 2001).

The use of wild relatives in genetic introgressions is generally accompanied by linkage drag associated with the introduction of unfavorable traits along with the favorable ones (Singh and Kumar, 2016), and this necessitates a significant and time-consuming breeding effort to recover the domesticated phenotype, particularly when the primary produce is the grain (Cox et al., 2018b; Habyarimana et al., 2019). Contrary to grain sorghum, however, where the kernel is the primary product, the aboveground biomass is the main target product in biomass sorghums and therefore the recovery of domestication-related plant ideotype traits from hybridizations, such as short-statured plants and big-sized grains are unnecessary in these sorghum types, implying the possibility for a faster recovery of perenniality conversion lines (Habyarimana et al., 2018). Although *S. propinquum* has also been crossed with domesticated *S. bicolor* to develop perennial genotypes (Kong et al., 2013, 2015), *S. halepense* is the preferred donor of this trait as it confers a stronger and more aggressive perenniality capable of withstanding freezing winters (Cox et al., 2002). *S. halepense* can be hybridized either with induced tetraploids or cytoplasmic-genetic male sterile diploids of *S. bicolor*, originating in both cases mainly tetraploid progenies (Piper and Kulakow, 2007; Nabukalu and Cox, 2016), although diploid descendants have also been observed (Dweikat, 2005; Cox et al., 2018a). Importantly, hybrid lines derived from *S. bicolor* × *S. halepense* crosses proved to be competitive with *S. bicolor* in terms of biomass production, opening up the possibility to straightforwardly develop perennial biomass sorghum cultivars (Habyarimana et al., 2018).

The selection for increasing sorghum biomass production can be either direct or indirect, i.e., targeted to different but correlated traits. However, as the biomass yield itself has generally a low heritability (Shiringani and Friedt, 2011), indirect selection was reported to have a comparable efficiency when correlated traits with higher heritability are used; moreover, such traits should be easier and cheaper to score than biomass yield, making indirect selection more cost-effective (Burks et al., 2015; Fernandes et al., 2018). In sorghum, several authors indicated plant height and maturity (Days to flowering), i.e., the number of days from sowing to 50% flowering, as the main determinants of biomass yield (Habyarimana et al., 2004; Upadhyaya et al., 2012, 2013; Kalpande et al., 2014), and can therefore be used for indirect selection for this trait. Plant height is the product of internode length and the number of nodes which are produced before flowering which, in turn, is a consequence of growth duration and the rate of internode production; therefore, besides being both correlated to yield, plant height and maturity are significantly correlated among themselves (Upadhyaya et al., 2012; Sadia et al., 2018). The dry mass fraction of fresh material represents one of the important biomass sorghum traits that determine the quality of the aboveground biomass produced (Habyarimana et al., 2016, 2018). Indeed, this plant characteristics is a key driver of the biofuel economics both at the bioreactor and logistics levels (Rentizelas, 2016), all of which motivates its use as selection criterion in sorghum breeding, with high biomass-yielding genotypes displaying high values of dry mass fraction of fresh material being preferred.

Plant height is traditionally reported to depend upon the action of four independent *Dw* dwarfing genes, *Dw1* to *Dw4*, having partial dominance for tallness and addictive effects (Quinby and Karper, 1954). So far, three of them have been isolated and cloned: *Dw1* (Sobic.009G230800) encodes for a putative membrane protein involved in the regulation of cell proliferation in the internodes (Hilley et al., 2016; Yamaguchi et al., 2016); the product of *Dw2* (Sobic.006G067600) is a protein kinase regulating stem internode length; and *Dw3* (Sobic.007G163800), which was the first to be identified, encodes a phosphoglycoprotein of the adenosine triphosphate-binding cassette (ABC) transporter superfamily involved in auxin transport, orthologous to maize *br2* (Multani et al., 2003). Although *Dw1-Dw4* explain most of the observed phenotypic variability for sorghum plant height, the existence of a fifth *Dw* gene (*Dw5*) has been recently reported (Chen et al., 2019); additionally, a number of other genes involved in gibberellin and brassinosteroid metabolism have been identified which can directly affect plant height (Ordonio et al., 2016b).

Six *Ma* (Maturity) loci, *Ma1-6*, are reported to control sorghum heading time (Quinby, 1967; Rooney and Aydin, 1999). Of these, *Ma1* is reported to have the largest effect; it encodes for the major flowering repressor, *SbPRR37* (PSEUDORESPONSE REGULATOR PROTEIN 37; Sobic.006G057866), which modulates the action of several floral inhibitors and activators (Murphy et al., 2011). The only *Ma4* has not yet been isolated, while *Ma2*, *Ma3*, *Ma5* and *Ma6* encode, respectively: a SET and MYND (SYMD) domain lysine methyltransferase (Sobic.002G302700) (Casto et al., 2019); a phytochrome B (Sobic.001G394400) (Childs et al., 1997); a phytochrome C (Sobic.001G087100) (Yang et al., 2014); and *Ghd7*, a CONSTANS, CO-like, and TOC1 (CCT) domain protein (Sobic.006G004400) (Murphy et al., 2014). All of them participate in a complex network of floral activators and repressors which, in ancestral sorghum genotypes evolved in tropical regions of Africa, functioned to inhibit flowering under long day conditions; loss-of-function mutations on *Ma* genes were selected during sorghum domestication to extend its cultivation in temperate zones. Other genes encoding mostly for transcription factors, participate in this regulatory network and contribute to the maturity trait (Ordonio et al., 2016b), although their effect is considered minor with respect to *Ma* genes.

Given the complex genetic base of typical quantitative and polygenic traits such as biomass yield and related traits, improving our knowledge on their genetic control is important to enhance sorghum breeding programs and the development of biomass sorghum cultivars. Uncovering quantitative trait loci (QTLs) explaining significantly sizeable variability in these complex traits can help expedite marker assisted traits introgression and the development of superior and/or farmer preferred cultivars. This area of research in genetics and molecular breeding is especially needed when traits introgression involves broadening the genetic base of cultivated sorghum with the use of wild relatives which provide an untapped source of useful alleles, but can have a detrimental linkage drag (Kumari et al., 2016) to select against. In this study, we aimed at investigating the genetic control of biomass yield and three biomass production-relevant traits ^_^ plant maturity, plant height, and dry mass fraction of fresh material ^_^ using a genome-wide association study (GWAS) approach in the genetic background of two distinct populations ^_^ a set of *S. bicolor* (Sb) landraces and breeding lines and a set of perennial *S. bicolor* × *S. halepense* (Sb × Sh) advanced (fixed) inbred lines derived from several parental lines ^_^ both of which make up a unique diversity panel in our breeding program and were amply described in our previous works (Habyarimana et al., 2016, 2018).

## Materials and Methods

### Plant Materials and Field Trials

The analyses were conducted on a diversity panel of 376 sorghum genotypes belonging to two distinct populations; the first, referred to as Sb, consisted of a *S. bicolor* population including 181 genotypes, mostly selections derived from landraces from Africa and Asia, and seven commercial hybrid lines included as controls. The second group, Sb × Sh, counted 188 advanced (fixed) recombinant inbred lines derived from several *S. bicolor* × *S. halepense* controlled hybridizations (single, double, and three-way crosses, and backcrosses) at different levels of filial progeny. Detailed information about the two populations can be found in previous publications (Habyarimana et al., 2016, 2018). Briefly, Sb × Sh genotypes were derived from annual/perennial (A/P) crosses, A/P backcrosses to annual recurrent parents (A^∗^2/P; BC1), perennial/perennial (P/P) and annual/perennial//perennial (A/P//P) crosses; with annual (A) parents being induced tetraploids (2*n* = 40), standard diploid (2*n* = 20), genetic male-sterile, and cytoplasmic-genetic male-sterile inbred *S. bicolor* lines, and perennial (P) parents consisting of either *S. halepense* or tetraploid hybrid lines obtained by crossing induced *S. bicolor* tetraploids with *S. halepense*. Open-field trials for the two populations were run in four consecutive years from 2014 to 2017 in the CREA Research Center for Cereal and Industrial Crops experimental station of Anzola (Bologna, Italy), using an augmented randomized complete block design (Federer, 1956) and commercial *S. bicolor* hybrids as controls. Crop management followed local extension services guidelines, as detailed in previous reports (Habyarimana et al., 2016, 2018).

### Phenotypic Data Collection

Four traits were evaluated following standard procedures: maturity or days to flowering (MAT), plant height (PH), dry mass fraction of fresh materials (DMC) and aboveground dry mass yield (DMY). Plant maturity was scored as the number of days between sowing and flowering, the latter being identified as the stage at which anthers were extruded in 50% of the plants from 50% of the spikelets on the panicle. Plant height was measured 1 week before harvest by estimating the mean height of the elementary plot using a 5 m telescopic rod. Plots were harvested using a single-row chopper harvester and a composite biomass sample of approximately 0.5–1 kg was collected for each genotype individually; the sample fresh weight was immediately measured, while the dry weight was determined after drying the sample at 80°C in a forced air oven for a few days, until weight was constant. The dry mass fraction of fresh material was calculated as the ratio of dry/fresh sample weights, and DMY was derived multiplying DMC by the fresh weight of the plot’s harvest, expressed in t/ha.

### Statistical Data Analysis

Statistical analyses were performed using the R language and environment (R Core Team, 2018). Correlation between different traits and different markers were assessed by the Pearson correlation coefficient. The significance of differences between phenotypic values for Sb and Sb × Sh populations was determined by Student’s *t*-test. Broad-sense heritability (*H*^2^) was estimated as repeatability (Gomez and Gomez, 1984) for each trait according to the following equation:

$$H^{2}=\frac{\sigma_{g}^{2}}{\sigma_{g}^{2}+\frac{\sigma_{e}^{2}}{n_{r}}}$$

with $\sigma_{g}^{2}$, $\sigma_{e}^{2}$, and *n*_*r*_ being genetic variance, residual variance and number of replications (years), respectively; variance components were estimated using the R package lme4, setting genotypes and replications (years) as random effects under the following linear mixed effect model:

$$y_{\text{ij}}=\mu +g_{\text{i}}+e_{\text{ij}}$$

where *y*_*ij*_ is the response for genotype *i* in replicate *j*, with *g* and *e* being the genotypic and residual (environmental) effects; in this work, yearly adjusted means were used as genotypic response. Polymorphism Information Content (PIC) was calculated for each SNP using the standard formula:

$$P\,I\,C=1-\sum_{i=1}^{n}p_{i}^{2}$$

where *p*_*i*_ is the frequency of the *ith* allele of the marker.

### DNA Extraction

To obtain plant material for DNA purification, 5–20 seeds per sample were sown in peat, watered, and treated with a fungicide and an insecticide (Ortiva, Syngenta, 1 ml/L and Confidor, Bayer, 0.75 ml/L) to protect young plantlets from pathogens and insects. Alternatively, seeds were treated with a seed-coating fungicide (Celest, Syngenta, 4 ml/L in water) and allowed to germinate on wet filter paper within petri dishes at a constant temperature of 25°C in a Venticell 111 incubator (MMM group) for 4–6 days. One to three healthy plantlets (nearly 10 cm tall) or 3–5 germinated seeds were collected for each sample and DNA was extracted using the GeneJET Plant Genomic DNA Purification Kit (Thermo Fisher Scientific), following manufacturer’s instructions. DNA concentration and purity were evaluated by a Tecan Infinite M200Pro spectrophotometer (Tecan Group Ltd., Switzerland), while DNA integrity was checked through 1% agarose gel electrophoresis with GelRed 10 μl/L (Biotium) as fluorescent dye. Aliquots of 60 μl at a concentration ≥ 10 ng/μl were prepared for each sample and used for downstream analyses.

### Genotyping-by-Sequencing

A genotyping-by-sequencing (GBS) strategy was adopted to obtain genotypic data from all the 376 samples. The methylation sensitive restriction enzyme ApeKI was used for library preparation, and sequencing was carried out on an Illumina HiSeq X Ten platform by BGI Hong Kong Company Limited. Two sequencing runs were performed, the first with 192 and the second with the remaining 184 samples, and sequence reads were aligned to the reference genome of *S. bicolor* version 3.1.1 (McCormick et al., 2018) to enable variants discovery. VCFtools^1^ (Danecek et al., 2011) was used to merge the two distinct matrices of 933,020 and 919,485 markers obtained from the two sequencing runs into a single dataset resulting in a total of 1,252,091 polymorphic loci. Using markers quality filters implemented in VCFtools, a working matrix of 92,383 high-quality SNPs to be used in association analyses was obtained by selecting biallelic SNPs only, minor allele frequency (MAF) ≥ 0.05, site quality or the Phred-scaled probability that reference/alternative alleles polymorphism exists at a given site data *Q* ≥ 40 (i.e., ≥99.99% base call accuracy), and missing genotypes (NA) ≤ 20%.

### Genome-Wide Association Study

Genome-wide association study was performed using the statistical genetics package Genome Association and Prediction Integrated Tool (GAPIT) (Tang et al., 2016) within the R environment (R Core Team, 2018). Missing data in the genotypic (SNP) matrix were imputed by Beagle (Browning et al., 2018); principal component analysis (PCA) and pairwise genetic relationship (kinship) matrix according to VanRaden (2008) were computed following the pipeline implemented in GAPIT; the kinship matrix and top three principal components were used in GWAS to control population and family structure. Two multi-locus GWAS algorithms were used to identify significant quantitative trait loci (QTLs) for the four traits under investigation: BLINK (Bayesian-information and Linkage-disequilibrium Iteratively Nested Keyway) is an evolution of FarmCPU (Fixed and random model Circulating Probability Unification) (Liu et al., 2016) improving statistical power and reducing computing time (Huang et al., 2018); and SUPER (Settlement of MLM Under Progressively Exclusive Relationship) relies on the FaST-LMM implementation of the Mixed Linear Model (MLM) to account for population structure and cryptic relationships, overcoming restrictions on the number of markers and increasing the statistical power (Wang et al., 2014). The distribution of observed vs. expected −log10(*p*) values was visualized using Quantile–Quantile (Q–Q) plots to test the fitness of GWAS models for all traits (Sharma et al., 2018); significant marker-trait associations, corresponding to putative QTLs for the four analyzed traits, were determined by the *P*-value less than 0.01/m, with m being the number of markers (Xu et al., 2018).

### Evaluation of Candidate Genes and Genomic Regions Linked to Major Effect Loci

The position of significant markers was compared to known QTLs for related traits, based on the information collected at the Sorghum QTL Atlas (Mace et al., 2018). The position of *Ma*, *Dw* and other genes known to be involved in maturity and plant height-related traits were obtained from published papers (Childs et al., 1997; Rooney and Aydin, 1999; Multani et al., 2003; Murphy et al., 2011, 2014; Yang et al., 2014; Hilley et al., 2016; Ordonio et al., 2016b; Yamaguchi et al., 2016; Casto et al., 2019) and their transcripts were identified on phytozome (Goodstein et al., 2012). Single nucleotide polymorphisms (SNPs) explaining more than 5% of the phenotypic variability of their associated traits were identified and their genomic regions were further analyzed in the process of functional GWAS. To perform functional GWAS, an interval of 500 Kb upstream and downstream the SNP position was considered, based on a genome-wide linkage disequilibrium (LD) decay cut-off at *R*^2^ = 0.1. Annotation details for genes within each region were retrieved using the Phytomine interface implemented in Phytozome (Goodstein et al., 2012).

## Results

### Phenotypic Variability, Heritability and Trait Correlation

**FIGURE 1:**
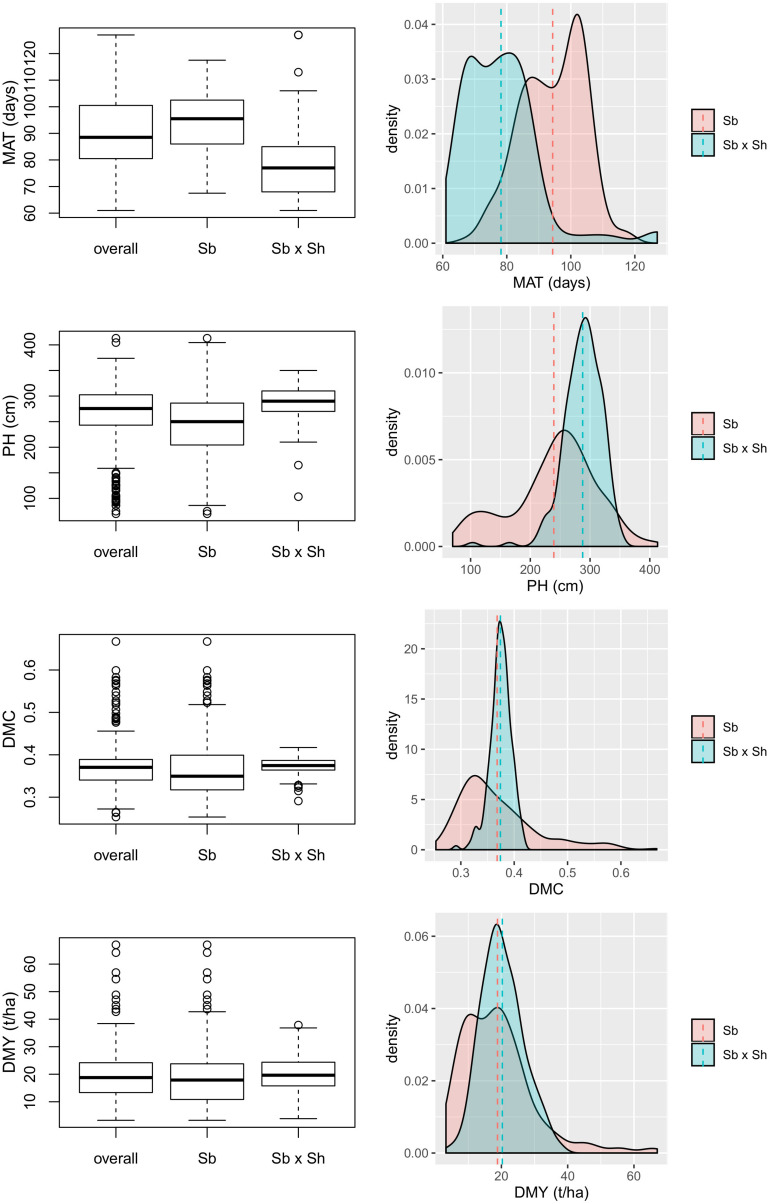
Variability of four analyzed traits depicted as box plots on the left and density plots on the right, comparing Sb and Sb × Sh populations; MAT, maturity; PH, plant height; DMC, dry mass fraction of fresh material; DMY, dry mass yield.

The variation of the four analyzed traits within the entire panel and the two populations separately is depicted in the box and density plots in Figure 1. The Sb × Sh lines showed a shorter flowering time (MAT: 78.2 vs. 94.3 days) and taller plants (PH: 287.6 vs. 239.3 cm) compared to Sb, and in both cases differences resulted highly significant (*p* = 2.2×10^−16^ and *p* = 2.7×10^−15^ for MAT and PH, respectively). No significant differences were registered among the two groups for dry mass fraction of fresh materials (DMC: 37.4 vs. 36.8%, *p* = 0.28) and yield (DMY: 20.2 vs. 18.8 t/ha, *p* = 0.11). Broad sense heritability (*H*^2^) was high for PH (0.93) and MAT (0.91), while it was medium for DMY (0.81) and DMC (0.63).

**FIGURE 2:**
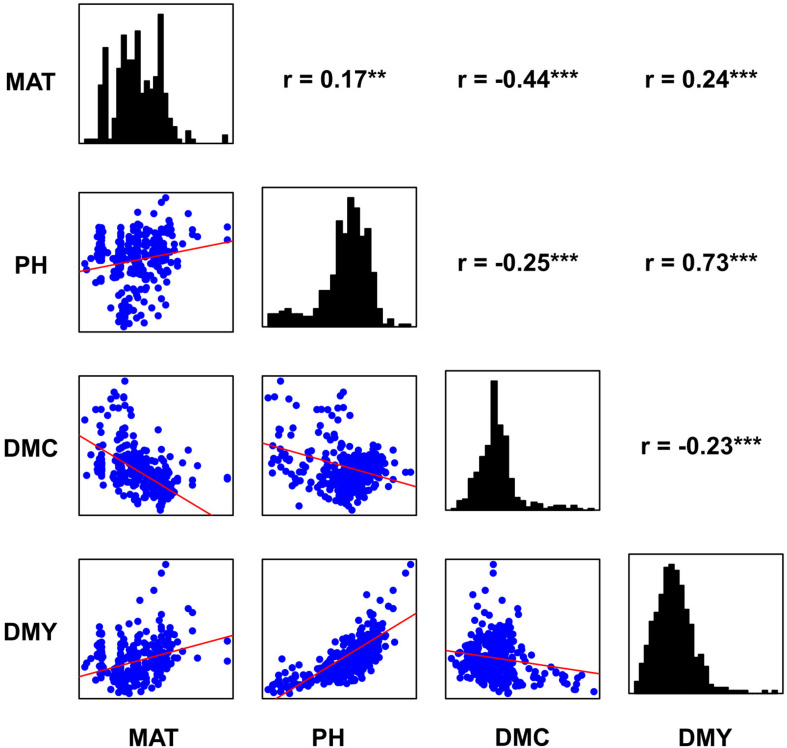
Distribution and correlation plots for the four traits; density histograms, scatter plots and Pearson correlation coefficients are shown, respectively, on the diagonal, lower left and upper right sides. **, *** Significant at the 0.01 and 0.001 probability levels, respectively.

Histograms, scatter plots and pairwise Pearson correlation coefficients for the four traits across the entire panel are reported in Figure 2. Dry mass fraction of fresh material (DMC) was negatively correlated to all other traits with low correlation coefficient (*r*) values ranging from −0.43 to −0.23. The remaining traits were positively correlated and showed *r* values ranging from low to medium according to the scale suggested in Habyarimana et al. (2020); the highest correlation was found between PH and DMY (*r* = 0.73). To investigate the effect of subpopulations on trait distribution and correlation, the Sb and Sb × Sh groups were analyzed separately; the obtained Pearson correlation coefficients are shown in Table 1 and the density plots are displayed in Figure 1. Correlations of opposite signs in the two populations were detected between PH and DMC (−0.34 in Sb vs. +0.22 in Sb × Sh) and DMC and DMY (−0.32 in Sb vs. 0.20 in Sb × Sh). On the other hand, MAT and DMY were correlated in Sb but not in Sb × Sh (0.47 in Sb vs. −0.05 in Sb × Sh). The correlation of MAT with PH was low in both Sb and Sb × Sh (*r* < 0.50), while the correlation of MAT with DMC was medium (*r* = −0.60) in Sb but low (*r* = −0.22) in Sb × Sh. Plant height maintained the highest positive correlation with DMY in both populations with medium values (*r* = 0.63 in Sb × Sh, and *r* = 0.79 in Sb) of the correlation coefficients.

**TABLE 1:** Pearson correlation coefficients between the four traits, calculated separately for the two populations: *S. bicolor* (Sb, above the diagonal) and *S. bicolor* × *S. halepense* (Sb × Sh, below the diagonal).

	MAT*	PH	DMC	DMY
MAT	–	0.413	−0.596	0.474
PH	0.190	–	−0.338	0.791
DMC	−0.216	0.223	–	−0.316
DMY	−0.051	0.631	0.203	–

*^∗^MAT, PH, DMC, DMY, respectively, maturity, plant height, dry mass fraction of fresh material, dry mass yield.*

### Population Structure and Allele Distribution

**FIGURE 3:**
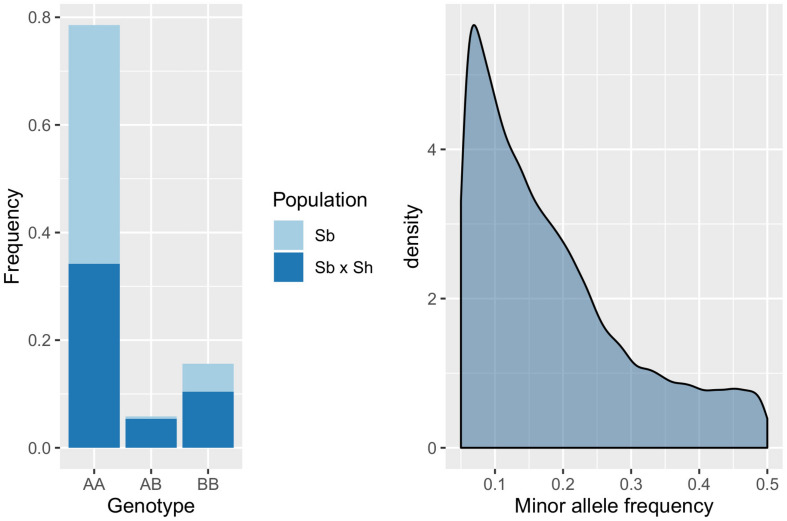
Distribution of SNP genotypes **(left)** and minor allele frequencies **(right)** in the evaluated populations; AA, AB, and BB indicate, respectively, homozygotes for the reference allele, heterozygotes and homozygotes for the alternative allele.

**FIGURE 4:**
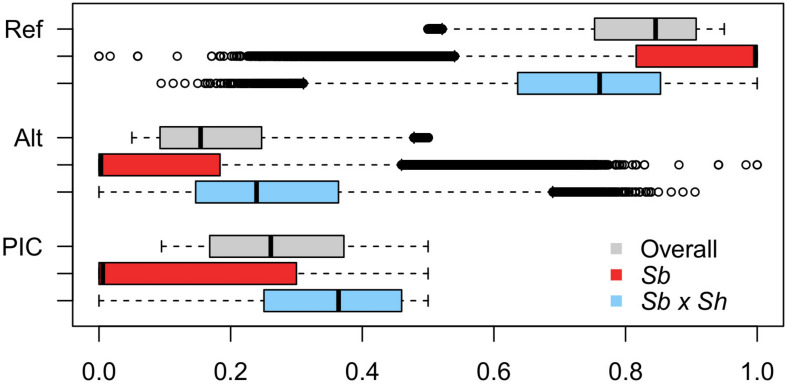
Boxplots depicting the distribution of reference (Ref) and alternative (Alt) allele frequencies, and PIC values across the whole dataset and in the two subpopulations separately.

**FIGURE 5:**
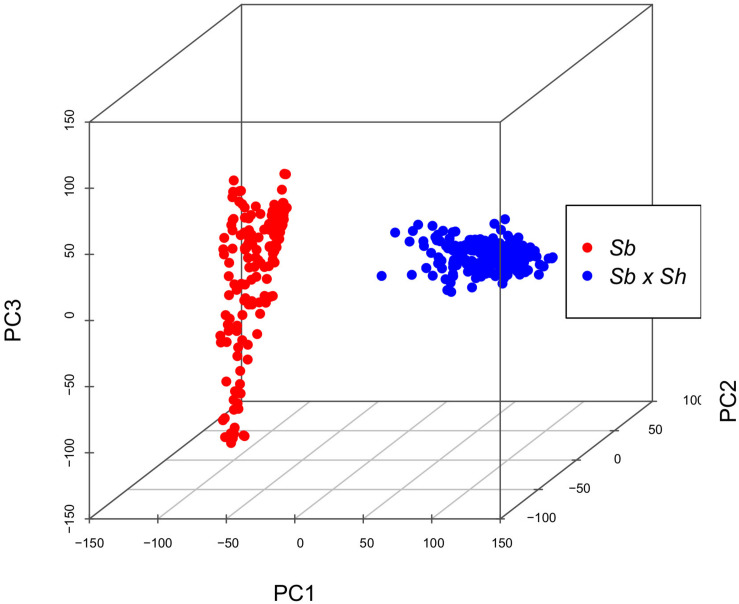
Three-dimensional principal component analysis based on SNP genotypic data.

Genotyping-by-sequencing (GBS) of the 376 samples yielded a raw data matrix consisting of 1,252,091 markers, that was filtered to obtain a dataset of 92,383 high quality biallelic SNPs (*Q* ≥ 40, MAF ≥ 0.05, missing data <20%). The distribution of SNP genotypes and minor allele frequencies (MAF) are reported in Figure 3. The frequency of heterozygotes was negligible within the Sb population, while it was higher in Sb × Sh; overall, however, heterozygotes were the rarest class in both populations, as required in order to have sufficient information and ensure statistical power for the GWAS approach. Figure 4 shows allele frequencies and PIC (polymorphic information content) values distribution in the analyzed populations; the degree of polymorphism resulted higher in Sb × Sh than in Sb, as indicated by the higher PIC values and frequency of alternative (minor) alleles. To better evaluate the informativeness of markers across the two populations, PIC values in Sb and Sb × Sh were plotted as a heatmap; the plot (Supplementary Figure 1) indicated the presence of SNPs with population specific PIC, but more SNPs were non-informative or poorly so in Sb relative to Sb × Sh. The filtered dataset, however, retained a satisfactory proportion of markers being highly informative in both populations. The population structure was analyzed by plotting the first 3 principal components (PC) and genomic relationship (kinship) matrix, as shown in Figure 5 and Supplementary Figure 3, respectively; both analyses clearly indicated that *S. bicolor* × *S.halepense* lines formed a distinct population from *S. bicolor* genotypes.

### Genome-Wide Marker-Trait Associations

The Genome-wide association study was conducted using two different algorithms, Blink and SUPER, which returned 8 and 66 significant SNP-trait associations, respectively, revealed by a total of 63 significant SNPs of which 55 were identified by SUPER algorithm. Quantile–Quantile plots reported in Supplementary Figure 3 show a good agreement between the expected and observed −log10(*p*) values using Blink, as reflected by low scores following the null hypothesis line, particularly for DMY and MAT traits; the SUPER method produced −log10(*p*) values relatively higher than expected, yet showing a greater statistical power as reflected by the higher number of significant associations detected.

**FIGURE 6:**
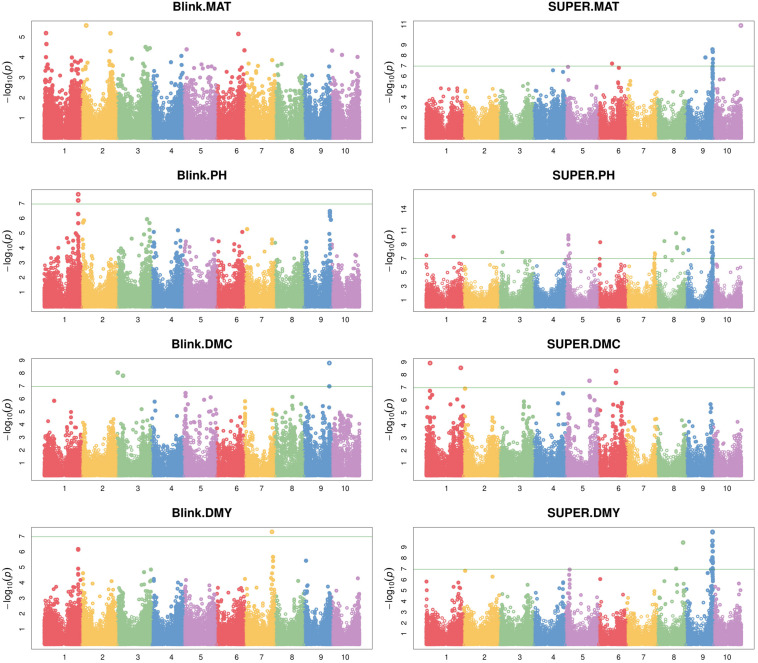
Manhattan plots representing the association between SNP markers and the four analyzed traits (from top to bottom: maturity, plant height, dry mass fraction of fresh material and dry mass yield) produced by algorithms Blink **(left)** and SUPER **(right)**. The solid horizontal line represents the genome-wide significance threshold as explained in the Section “Materials and Methods.”

The complete list of markers significantly associated with phenotype is reported in Table 2 and the GWAS output is graphically depicted in Manhattan plots (Figure 6). A total of 63 significant SNPs were located on 8 of the 10 sorghum chromosomes, with chromosomes 2 and 4 showing no associations, while chromosome 9 (Chr 9) showed the highest number of associations (27 markers associated with all the four traits). Plant maturity was associated with 7 SNPs identified by SUPER algorithm and located on chromosomes 6, 9, and 10. Plant height recorded the highest number of associated SNPs: 42 spanning 7 chromosomes (1, 3, 5, 6, 7, 8, and 9), 4 of which (all on Chr 1) were detected by Blink and the remaining by SUPER. Dry mass fraction of fresh material was associated with 8 markers distributed on chromosomes 1, 3, 5, 6, and 9; five and three of these markers were identified by SUPER and Blink, respectively. Dry mass yield was associated with seventeen SNPs located on chromosomes 7, 8, and 9, with the only marker on Chr 7 being detected by the Blink algorithm. Eleven markers, two on Chromosomes 8 and 9 on Chr 9, were associated, each, to two different traits reflecting probable pleiotropic situations. Of these SNPs, two located on Chr 9 (Chr9_58408977 and Chr9_58527007) were significantly associated with maturity and dry mass yield, while the others (Chr8_41653835, Chr8_56708385, Chr9_57470027, Chr9_57601584, Chr9_57601601, Chr9_57716476, Chr9_57807056, Chr9_57856468, and Chr9_57919263) were associated with plant height and dry mass yield. The majority of significant SNPs (44 out of 63) were polymorphic in both Sb and Sb × Sh populations, while 16 and 3 were specific for the *S. bicolor* and the *S. bicolor* × *S. halepense* populations, respectively.

**TABLE 2:** Significant marker/trait associations identified by GWAS analysis; for each marker, are reported alleles, genomic location (chromosome and position in bp), GWAS method which led to the identification of a significant association, trait of interest, *p*-value, frequencies of the reference (Ref) and alternative (Alt) alleles, effect of the Alt allele, population in which the polymorphism was detected, and *R*^2^(%) values.

SNP	Alleles	Chr	Position	Method	Trait	*P-value*	Ref	Alt	Effect	Polymorphism	*R*^2^ (%)
Chr1_1022486	T/C	1	1,022,486	SUPER	PH	3.9E-08	0.83	0.17	−0.139	Sb	0.6
Chr1_8820891*	T/C	1	8,820,891	SUPER	DMC	1.2E-09	0.92	0.08	−0.208	Sb/Sb × Sh	16.2
Chr1_59791089	A/G	1	59,791,089	SUPER	PH	8.0E-11	0.92	0.08	0.173	Sb/Sb × Sh	3.2
Chr1_73847018	A/C	1	73,847,018	Blink	PH	2.4E-08	0.74	0.26	−0.315	Sb/Sb × Sh	2.6
Chr1_73855085	C/T	1	73,855,085	Blink	PH	6.2E-08	0.72	0.28	−0.305	Sb/Sb × Sh	3.6
Chr1_73855086	T/G	1	73,855,086	Blink	PH	6.2E-08	0.72	0.28	−0.305	Sb/Sb × Sh	3.6
Chr1_73855096	C/G	1	73,855,096	Blink	PH	6.2E-08	0.72	0.28	−0.305	Sb/Sb × Sh	3.6
Chr1_75442083	C/A	1	75,442,083	SUPER	DMC	2.9E-09	0.94	0.06	0.089	Sb/Sb × Sh	2.1
Chr3_948375	T/A	3	948,375	Blink	DMC	9.1E-09	0.93	0.07	0.413	Sb/Sb × Sh	0.1
Chr3_6805616	C/A	3	6,805,616	SUPER	PH	1.4E-08	0.85	0.15	−0.109	Sb/Sb × Sh	<0.1
Chr3_12314731*	G/T	3	12,314,731	Blink	DMC	1.6E-08	0.88	0.12	−0.398	Sb	7.8
Chr5_6800612	T/C	5	6,800,612	SUPER	PH	1.3E-10	0.63	0.37	−0.078	Sb/Sb × Sh	0.3
Chr5_6800639	C/T	5	6,800,639	SUPER	PH	1.9E-10	0.65	0.35	−0.064	Sb/Sb × Sh	0.3
Chr5_6800653	G/T	5	6,800,653	SUPER	PH	4.7E-08	0.66	0.34	0.028	Sb/Sb × Sh	4.2
Chr5_6800722	T/A	5	6,800,722	SUPER	PH	5.1E-11	0.63	0.38	−0.075	Sb/Sb × Sh	0.3
Chr5_6903496	G/A	5	6,903,496	SUPER	PH	1.2E-09	0.78	0.22	0.057	Sb/Sb × Sh	0.2
Chr5_10479364	C/G	5	10,479,364	SUPER	PH	1.9E-08	0.77	0.23	−0.100	Sb/Sb × Sh	<0.1
Chr5_52531559	C/A	5	52,531,559	SUPER	DMC	3.0E-08	0.91	0.09	0.041	Sb	2.0
Chr6_3888329	G/A	6	3,888,329	SUPER	PH	5.0E-10	0.87	0.13	−0.146	Sb	1.1
Chr6_29554814	T/C	6	29,554,814	SUPER	MAT	6.2E-08	0.86	0.14	−0.071	Sb × Sh	0.1
Chr6_37905197	C/T	6	37,905,197	SUPER	DMC	4.4E-08	0.84	0.16	0.096	Sb/Sb × Sh	0.6
Chr6_38393064	T/G	6	38,393,064	SUPER	DMC	5.1E-09	0.85	0.15	0.035	Sb/Sb × Sh	0.5
Chr7_58880630	G/C	7	58,880,630	Blink	DMY	5.2E-08	0.92	0.08	0.414	Sb	0.1
Chr7_59810341	A/G	7	59,810,341	SUPER	PH	6.7E-17	0.68	0.32	−0.076	Sb/Sb × Sh	0.4
Chr7_60308754	C/T	7	60,308,754	SUPER	PH	6.2E-08	0.77	0.23	−0.027	Sb/Sb × Sh	<0.1
Chr7_60741571	G/A	7	60,741,571	SUPER	PH	1.9E-08	0.60	0.40	0.022	Sb/Sb × Sh	<0.1
Chr7_61501575	G/C	7	61,501,575	SUPER	PH	2.7E-08	0.80	0.20	0.083	Sb	0.2
Chr7_61501583	C/G	7	61,501,583	SUPER	PH	2.7E-08	0.80	0.20	0.083	Sb	0.2
Chr8_16157163	G/A	8	16,157,163	SUPER	PH	3.5E-10	0.95	0.05	0.144	Sb	2.6
Chr8_18075160	T/A	8	18,075,160	SUPER	PH	5.9E-08	0.95	0.05	0.207	Sb	2.2
Chr8_41653835*	G/A	8	41,653,835	SUPER	DMY	9.6E-08	0.95	0.05	0.124	Sb	6.9
				SUPER	PH	2.5E-11			0.186		3.3
Chr8_43617085	T/A	8	43,617,085	SUPER	PH	2.4E-09	0.94	0.06	0.136	Sb	2.5
Chr8_43754007	G/A	8	43,754,007	SUPER	PH	3.8E-09	0.95	0.05	0.178	Sb	2.7
Chr8_56708385*	G/T	8	56,708,385	SUPER	DMY	3.7E-10	0.94	0.06	0.050	Sb	7.1
				SUPER	PH	1.9E-08			0.158		1.8
Chr8_57785385	C/T	8	57,785,385	SUPER	PH	1.4E-10	0.95	0.05	0.124	Sb	3.2
Chr9_5710971	G/A	9	5,710,971	SUPER	PH	6.8E-09	0.90	0.10	−0.209	Sb/Sb × Sh	1.0
Chr9_42350413	C/T	9	42,350,413	SUPER	MAT	1.6E-08	0.85	0.15	−0.035	Sb × Sh	<0.1
Chr9_54918217	C/T	9	54,918,217	Blink	DMC	1.6E-09	0.90	0.10	0.377	Sb	3.3
Chr9_55056612	A/G	9	55,056,612	SUPER	DMY	1.6E-08	0.95	0.05	−0.221	Sb/Sb × Sh	4.6
Chr9_55076405	T/A	9	55,076,405	SUPER	PH	1.4E-08	0.89	0.11	−0.106	Sb/Sb × Sh	1.4
Chr9_56196252	C/T	9	56,196,252	SUPER	DMY	2.0E-08	0.89	0.11	−0.221	Sb/Sb × Sh	1.2
Chr9_56475857	T/C	9	56,475,857	SUPER	PH	9.7E-08	0.58	0.43	−0.053	Sb/Sb × Sh	0.1
Chr9_56496841	A/C	9	56,496,841	SUPER	DMY	1.4E-08	0.93	0.07	−0.042	Sb/Sb × Sh	4.7
Chr9_57420844	A/G	9	57,420,844	SUPER	PH	5.3E-09	0.94	0.06	−0.049	Sb/Sb × Sh	2.8
Chr9_57470027	G/A	9	57,470,027	SUPER	DMY	7.7E-10	0.95	0.05	−0.219	Sb/Sb × Sh	3.4
				SUPER	PH	8.4E-11			−0.179		1.7
Chr9_57601584*	G/A	9	57,601,584	SUPER	DMY	6.5E-10	0.92	0.08	−0.052	Sb/Sb × Sh	5.9
				SUPER	PH	1.3E-11			−0.088		4.4
Chr9_57601601*	G/C	9	57,601,601	SUPER	DMY	8.6E-09	0.90	0.10	−0.034	Sb/Sb × Sh	5.2
				SUPER	PH	5.5E-10			−0.059		4.1
Chr9_57684325	C/T	9	57,684,325	SUPER	PH	1.3E-08	0.94	0.06	−0.109	Sb/Sb × Sh	1.6
Chr9_57687430	T/C	9	57,687,430	SUPER	MAT	2.7E-09	0.83	0.17	−0.068	Sb/Sb × Sh	2.7
Chr9_57716476	G/A	9	57,716,476	SUPER	DMY	2.6E-10	0.92	0.08	−0.154	Sb/Sb × Sh	3.4
				SUPER	PH	5.4E-08			−0.129		1.6
Chr9_57807056	A/G	9	57,807,056	SUPER	DMY	1.2E-08	0.94	0.06	−0.173	Sb/Sb × Sh	2.6
				SUPER	PH	2.4E-09			−0.087		1.7
Chr9_57856468	G/A	9	57,856,468	SUPER	DMY	3.8E-11	0.93	0.07	−0.151	Sb/Sb × Sh	3.9
				SUPER	PH	2.4E-09			−0.150		1.9
Chr9_57919263	G/A	9	57,919,263	SUPER	DMY	4.2E-11	0.93	0.07	−0.214	Sb/Sb × Sh	3.7
				SUPER	PH	5.0E-09			−0.170		1.5
Chr9_57938398	T/C	9	57,938,398	SUPER	DMY	8.6E-08	0.93	0.07	−0.134	Sb/Sb × Sh	2.7
Chr9_57956804	T/C	9	57,956,804	SUPER	PH	6.5E-08	0.93	0.07	−0.080	Sb/Sb × Sh	1.3
Chr9_57956805	C/A	9	57,956,805	SUPER	PH	8.1E-08	0.93	0.07	−0.078	Sb/Sb × Sh	1.3
Chr9_58293125	T/A	9	58,293,125	SUPER	DMY	2.4E-09	0.75	0.25	−0.108	Sb/Sb × Sh	0.9
Chr9_58408977	G/A	9	58,408,977	SUPER	DMY	2.9E-08	0.94	0.06	−0.196	Sb/Sb × Sh	2.7
				SUPER	MAT	4.6E-09			−0.064		4.5
Chr9_58527007*	C/T	9	58,527,007	SUPER	DMY	2.1E-08	0.93	0.07	−0.236	Sb/Sb × Sh	2.1
				SUPER	MAT	5.5E-08			−0.047		5.4
Chr9_58584246*	G/A	9	58,584,246	SUPER	MAT	2.3E-08	0.94	0.06	0.021	Sb/Sb × Sh	5.9
Chr9_58811494	G/C	9	58,811,494	SUPER	PH	1.9E-08	0.81	0.19	0.134	Sb	0.4
Chr9_59310281	A/G	9	59,310,281	SUPER	PH	4.1E-08	0.51	0.49	0.008	Sb/Sb × Sh	2.0
Chr10_59784957	C/T	10	59,784,957	SUPER	MAT	1.3E-11	0.94	0.06	0.175	Sb × Sh	3.4

*^∗^Markers with highest effect on their associated trait; threshold set at R^2^ ≥ 0.05.*

Most of the detected polymorphisms had a minor effect on their associated traits as reflected by the low values of the coefficient of determination (*R*^2^) reported in Table 2. Eight significant markers on chromosomes 1, 3, 8, and 9 showed *R*^2^≥5% for dry mass fraction of fresh material (Chr1_8820891 and Chr3_12314731), dry mass yields (Chr8_41653835, Chr8_56708385, Chr9_57601584, and Chr9_57601601), and maturity (Chr9_58527007 and Chr9_58584246); all but one (Chr3_12314731 identified by Blink) of these markers were identified using SUPER algorithm. All of the eight markers were polymorphic in Sb and Sb × Sh populations except three (Chr3_12314731, Chr8_41653835, and Chr8_56708385) that were polymorphic only in Sb population. The only marker with a major effect (*R*^2^≥15%) according to the scale defined in Habyarimana et al. (2019), was Chr1_8820891, explaining 16.2% of the phenotypic variability observed in dry mass fraction of the fresh material. In most SNP-trait associations (48 out of 74), the Alt allele had a negative effect on the trait of interest.

### Pairwise Statistical Association Among Significant SNPs

**FIGURE 7:**
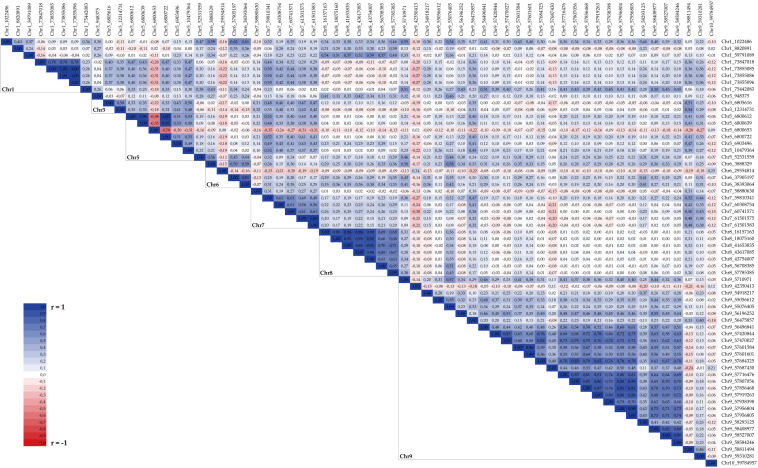
Pairwise correlation coefficients between GWAS significant SNPs.

Several blocks of highly correlated markers, likely belonging to a common haplotype, were identified; Figure 7 reports pairwise Pearson coefficients (*r*) for all significant markers identified by GWAS. On Chr 1, four SNPs (Chr1_73847018, Chr1_73855085, Chr1_73855086, and Chr1_73855096) were highly associated, with *r*≥0.78, and covered a very narrow region at 73.85 Mbp. Two out of three significant SNPs on Chr 3 (Chr3_6805616 and Chr3_12314731) showed a medium correlation (*r* = 0.58) despite being physically distant at 5.5 Mbp. On Chr 5, three out of four markers located at 6.8 Mbp (Chr5_6800612, Chr5_6800639, and Chr5_6800722) were very highly associated (*r*≥0.95) while the fourth (Chr5_6800653) showed a negative correlation with them (*R* ≤ −0.55), likely identifying an alternative haplotype at the same locus; two other SNPs located in close proximity (Chr5_6903496 and Chr5_10479364) were correlated with the main block with *r*≥0.50. Two markers were associated on Chr 6 (Chr6_37905197 and Chr6_38393064, *r* = 0.81), while on Chr 7 six significant SNPs spanning a 2.6 Mbp region were positively correlated: *r*≥0.61 was found between Chr7_59810341, Chr7_60308754, and Chr7_60741571, and between Chr7_60741571, Chr7_61501575, and Chr7_61501583. All significant markers on Chr 8 were positively correlated among themselves, with pairwise *r*≥0.62, despite being distantly mapped (from 16.2 to 57.9 Mbp) on the chromosome. Finally, within the main hotspot of significant markers on Chr 9 different blocks of associated SNPs were detected; particularly, markers placed between Chr9_57420844 and Chr9_58584246 formed a large block with a generally high positive correlation ranging from 0.49 to 0.99 with the exception of two SNPs (Chr9_57687430 and Chr9_58293125) showing lower values of the correlation coefficients with the other SNPs and among themselves.

### Location of Significant SNPs Relative to Known Genes/QTLs for the Plant Characteristics of Interest

**FIGURE 8:**
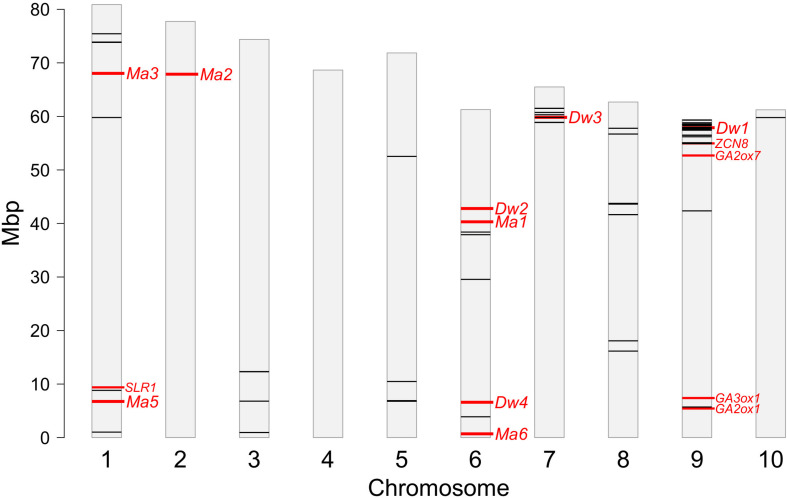
Genomic positions of SNPs (black lines) significantly associated to one or more traits after GWAS; the locations of major genes controlling maturity (*Ma*) and plant height (*Dw*) and other candidate genes involved in the same traits are reported in red; refer to the main text for further details.

Several markers were located in close proximity of known genes and QTLs controlling plant height or maturity, as shown in Figure 8. Two main hotspots of significant SNPs co-located with genes *Dw1* and *Dw3* toward the end of chromosomes 9 and 7, respectively. The hotspot on Chr9 covered the region from 54.9 to 59.3 Mbp and included 25 SNPs significantly associated to all the four traits (Table 2 and Figure 8), 16 of which located within a distance < 1 Mbp from *Dw1* (57.1 Mbp) (Hilley et al., 2016; Yamaguchi et al., 2016). The same region also harbors two candidate genes for gibberellin (GA) metabolism (*SbGA2ox7*, 54.7 Mbp) and heading date (*SbZCN8*, 55.0 Mbp) (Ordonio et al., 2014, 2016b); three SNPs ^_^ Chr9_54918217, Chr9_55056612, and Chr9_55076405 ^_^ significantly associated with DMC, DMY, and PH, respectively, lay into a very narrow interval at <120 Kbp from *SbZCN8* and <380 Kbp from *SbGA2ox7* (Table 2 and Figure 8). Another significant SNP on Chr 9 (Chr9_5710971 associated with PH) fell in close proximity of the GA-related genes *SbGA2ox1* (at 270 Kbp) and *SbGA3ox1* (1.2 Mbp) (Ordonio et al., 2014, 2016b). The hotspot on Chr 7 included six SNPs significantly associated with PH (5 SNPs) and DMY (1 SNP) and positioned from 58.9 to 61.5 Mbp, with marker Chr7_59810341 being only 20 Kbp upstream *Dw3* (59.8 Mbp) (Multani et al., 2003). No significant associations were found in proximity of *Dw2* and *Dw4*.

The position of *Ma* genes did not match with significant SNPs detected by GWAS (Figure 8); the two markers (Chr6_38393064 and Chr1_8820891 associated with DMC) closest to the *Ma* genes were found at 2 Mbp from *Ma1* located at 40.3 Mbp on Chr 6 (Murphy et al., 2011), and *Ma5* located at 6.75 Mbp on Chr 1 (Yang et al., 2014). However, the SNP Chr1_8820891 was much closer to another candidate gene, *SbSLR1* located at 9.4 Mbp on Chr 1 and involved in GA signaling and plant height regulation (Ordonio et al., 2016b).

**FIGURE 9:**
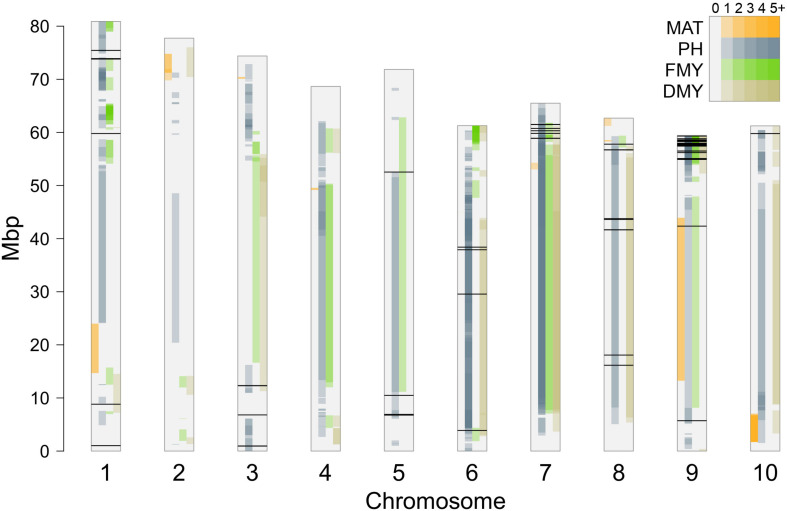
Position of significant SNPs compared to known QTLs reported for maturity (MAT), plant height (PH), fresh (FMY) and dry (DMY) mass yields derived from the Sorghum QTL Atlas (Mace et al., 2018); color intensity inside each bar reflects the number of QTL confidence intervals harboring each SNP position.

The positions of significant SNPs detected by GWAS were compared to genomic locations of known QTLs for maturity (including duration of vegetative stage and photoperiod sensitivity), plant height, fresh and dry biomass production, retrieved from the Sorghum QTL Atlas^2^ (Mace et al., 2018), as shown in Figure 9. Many QTLs have been reported from several studies, and spread along all the 10 sorghum chromosomes. Of the 63 significant SNPs reported in this work, only three SNPs on chromosomes 1 and 3 (Chr1_1022486, Chr1_59791089, and Chr3_6805616) that happened to be associated with PH, did not fall into confidence intervals of any known QTL.

### Identification of Genomic Regions and Transcripts Containing SNPs With Major (*R*^2^≥5%) Breeding Interests

The analysis of the genome-wide linkage disequilibrium decay with a cut-off at *r*^2^ = 0.1 suggested a region of approximately 500 Mb surrounding each significant SNP as the most likely to harbor genes linked with the SNP and determining the traits of interest, consistently with a previous study on a subset of the same population (Habyarimana et al., 2019). Eight SNPs explaining more than 5% of the variability in their associated trait were detected (Table 2), identifying six distinct genomic regions on four chromosomes. The Supplementary Table 1 lists the genes annotated in these regions, indicating their physical distance from significant markers and ontology annotations. On chromosome 1, the region spanning 1 Mb around marker Chr1_8820891 associated to DMC, contains 135 transcripts reported on the Sorghum reference genome sequence; marker Chr3_12314731, that is also associated to DMC, identified a region containing 70 genes. Two regions associated to DMY were identified on chromosome 8: the first, identified by SNP Chr8_41653835, contained only 5 transcripts, while the second, centered on Chr8_56708385, showed the presence of 76 genes. Finally, four putative major-effect SNPs (Chr9_57601584 and Chr9_57601601 associated to DMY; Chr9_58527007 and Chr9_58584246 associated to MAT) fell within the main hotspot on chromosome 9, identifying a region spanning nearly 2 Mb between 57 and 59 Mbp, which contained 263 genes (Supplementary Table 1). Among the SNPs selected on chromosome 9, Chr9_57601584 and Chr9_57601601 identify a single locus, being only 17bp distant from each other; the distance between Chr9_58527007 and Chr9_58584246 is also very narrow, implying that the two SNPs are linked and probably co-inherited during meiosis.

Three of the SNPs (Chr1_8820891, Chr8_41653835 and Chr9_58584246) selected as being of major breeding interests are located in intergenic regions, while the remaining five fell within the sequence of a gene/transcript. Specifically, Chr3_12314731 (associated to DMC) is located in the first intron of transcript Sobic.003G131300; Chr8_56708385 (associated with DMY) falls within the coding sequence of Sobic.008G138100; the two markers Chr9_57601584 and Chr9_57601601 (DMY) are located in the second exon of Sobic.009G237900; finally, Chr9_58527007 (associated with MAT) fell in the 3′ untranslated region of two possible transcripts: Sobic.009G249900 and Sobic.009G250000. Of the three SNPs located in coding regions, two correspond to silent mutations while one (Chr9_57601584, G to A) causes a semi-conservative amino acid change (Alanine to Valine) at position 238 out of 259 of the putative protein.

### Identification of Candidate Genes on the Reference Genome Sequence

The gene closest to the major effect marker Chr1_8820891 explaining 16.2% of the variation for dry mass fraction of fresh material, is Sobic.001G112500, a putative zinc-finger homeodomain (ZF-HD) protein, a class of transcription factors in which the HD domain binds DNA, and the ZF domain can enhance the protein–DNA interaction (Hu et al., 2008). The second major breeding interest SNP for DMC, Chr3_12314731 (*R*^2^ = 7.8%), is located in the first intron of gene Sobic.003G131300 whose function is not annotated yet, although it is reported to be differentially downregulated in upper vs. lower vegetative leaf whorl. Two major breeding interest markers on chromosome 8 had significant effects on DMY; the first, Chr8_41653835 (*R*^2^ = 6.9%), falls in a pericentromeric region with a low gene density; only 5 genes are harbored in the 1 Mb interval centered on the SNP position, two of which (including the closest one, Sobic.008G092866) are likely associated to transposable elements. The second SNP, Chr8_56708385 (*R*^2^ = 7.1%), falls within the coding sequence of Sobic.008G138100, whose predicted protein product shows similarities with the exostosin family.

Four major breeding interest SNPs were found within the hotspot on chromosome 9; transcript Sobic.009G237900 contains two of them, Chr9_57601584 and Chr9_57601601, associated to DMY with *R*^2^ = 5.9% and 5.5%, respectively. The predicted product of Sobic.009G237900 is a putative plastocyanin which is a protein involved in the photosynthetic electron transport chain between PSII and PSI (Sato et al., 2003). The remaining two major effect SNPs on chromosome 9 are associated to plant maturity: the physical distance between Chr9_58527007 (*R*^2^ = 5.4%) and Chr9_58584246 (*R*^2^ = 5.9%) is 57 Kb, indicating that they might be associated to the same functional gene; this gene dense region includes 10 transcripts, several of which have a possible functional correlation with maturity and biomass-related traits (Supplementary Table 1). For instance, Sobic.009G249900 is the putative ortholog of rice jasmonic acid-amido synthetase *JAR1*, which modulates light and JA signaling in the photomorphogenesis of rice and is involved in plant response to several stresses (Riemann et al., 2008; Wakuta et al., 2011; Svyatyna et al., 2014); Sobic.009G250000 is a putative transcription factor of the basic Helix–Loop–Helix (bHLH) family; Sobic.009G250100 and Sobic.009G250500 lack functional annotations, but for the former a probable zinc-ribbon domain is reported suggesting a possible role as transcription factor; Sobic.009G250200 encodes a putative transmembrane amino acid transporter whose expression is down-regulated in response to drought stress (Abdel-Ghany et al., 2020); Sobic.009G250300 is homolog to rice OsPAP10c, a secreted purple acid phosphatase (PAP) that by scavenging organic phosphorus (P) in the rhizosphere enhances the plant utilization efficiency of external organic P (Lu et al., 2016); the product of Sobic.009G250400 has sequence homology to SIG5, a nuclear encoded σ^70^ subunit of plastidial RNA polymerase that drives chloroplast transcriptional response to light intensity and the circadian clock (Belbin et al., 2017); Sobic.009G250600 encodes a F-box protein and its role might therefore be related to protein degradation via the ubiquitin -proteasome pathway, with a wide variety of possible cytological, physiological and developmental effects (Zhang et al., 2019); Sobic.009G250700 corresponds to a pentatricopeptide repeat (PPR) protein, a large family typically involved in the modulation of organellar gene expression, organelle biogenesis and function, with considerable effects on photosynthesis, respiration, plant development and environmental responses (Barkan and Small, 2014); finally, Sobic.009G250800, the gene closest to SNP Chr9_58584246 (55 bp distant), encodes for a putative pectin lyase reported to be up-regulated in response to heat stress (Johnson et al., 2014).

## Discussion

In this study a diversity panel of *Sorghum bicolor* (Sb) and advanced inbred lines derived from the interspecific hybridization *S. bicolor* × *S. halepense* (Sb × Sh) were evaluated genotypically and phenotypically for biomass yield and biomass production relevant traits. Understanding the genetic base of traits and their correlations is important to improve selection efficiency, especially for those quantitatively inherited traits, like biomass yield for which indirect selection for correlated traits with higher heritability can be useful by expediting the cultivar development process and making breeding for biomass yields more cost-effective. As expected, dry mass yield was significantly influenced by environmental factors as reflected by lower heritability compared with maturity and plant height. In the diversity panel evaluated in this work, plant height and maturity are more suitable targets for marker-assisted selection and indirect selection for biomass yields as they displayed high heritability. The use of *S. halepense* genome in this study was valuable in several respects. On the one hand, while many previous studies contributed to elucidating the genetic control of biomass-related traits in *S. bicolor*, the possible contribution of *S. halepense* remained largely unexplored. Besides its ability to confer perenniality to hybrids thanks to overwintering rhizomes, its effect on other traits has been poorly investigated owing probably to the fact that the mainstream in the breeding community considers that this species transmits mainly weediness-related unfavorable traits. However, recent reports highlighted *S. halepense* as a possible source of useful alleles even for traits other than perenniality, such as the content in grain antioxidants (Habyarimana et al., 2019). It is therefore important to better characterize the untapped genetic potential of *S. halepense* in breeding, especially for biomass sorghum, and this work was undertaken for this purpose and to fill-in the above-mentioned gaps.

Structure analyses showed that Sb and Sb × Sh populations were genetically different, and corrective measures were necessary to correct for population structure and avoid false positive associations. In this study, fixed genotypes (lines and landraces) were included in the diversity panel and belonged to *S. bicolor* and *S. bicolor* × *S. halepense* crosses (single, double, and three-way crosses, and backcrosses) involving several parental lines, meaning that GWAS investigation was justified as in previous studies (e.g., Yuan et al., 2019). The observed population structure implied the existence of genetic relationships among individuals in the populations, and this can give rise to confounding effects. However, implemented corrective measures to model and hence, correct for these confounding genetic relationships by using not only the algorithms implementing mixed modeling, but also including in the GWAS models the principal component analysis and kinship matrix as covariates (Sul et al., 2018; Habyarimana et al., 2019; Yuan et al., 2019).

Differences between Sb and Sb × Sh populations emerged clearly both from phenotypic and genotypic data. The limited number of parents used in hybridizations (Habyarimana et al., 2018) is reflected by the narrow variation of all the analyzed traits in Sb × Sh compared to Sb (Figure 1). While dry mass fraction of fresh material (DMC) and dry mass yield (DMY) showed comparable means between the two populations, significant differences were found for the number of days to maturity (MAT) and plant height (PH). These latter two traits are generally positively correlated: a longer vegetative stage entails a longer growth period and a higher numbers of internodes developed before the plant shifts to the flowering stage (Upadhyaya et al., 2012; Sadia et al., 2018). In our case, however, the Sb × Sh genotypes matured earlier but were taller than Sb (Figure 1). As a result, the correlation between MAT and PH resulted very low when analyzed in the entire pool of genotypes in the diversity panel (Figure 2), but it increased when only the Sb population was taken into account (Table 1). On the other hand, MAT was negatively correlated to DMC, meaning that lines maturing earlier were drier at harvest (Table 1). We also observed a weak correlation between MAT and the other traits, particularly biomass yield, within the Sb × Sh group (Figure 1), implying that plant maturity can be genetically manipulated without compromising biomass yields and quality (dry mass fraction of fresh material) in the Sb × Sh population. Like most weeds, *S. halepense* flowers quite rapidly after emergence, which enables a fast seed development and dispersal (Monaghan, 1979); this trait was transmitted to the recombinant inbred lines, which, however, contrary to what would be expected in Sb population, performed well in terms of plant height and biomass yield (Habyarimana et al., 2018).

As expected, differences between the two populations became striking when genotypic data were analyzed. A population structure composed of two well distinct subgroups is evident both from the kinship matrix and principal component analysis (Figure 5 and Supplementary Figure 2). The Sb × Sh population showed a greater degree of polymorphism than Sb as reflected by the higher values of the PIC and the increased frequency of alternative SNP alleles and heterozygotes in this population (Figure 4). This finding is consistent with previous analyses carried out on a small subset of the same two populations (Habyarimana et al., 2019), and can be explained by considering the different composition of the two subgroups. *S. bicolor* has undergone the bottleneck of domestication, which generally implies a narrowing of the genetic base with respect to wild species. However, the tetraploid status of *S. halepense* and its descendants may have played a major role in shaping the allelic composition in the Sb × Sh population. First of all, it should be considered that fixation of alleles requires a higher number of generations in polyploids, and heterozygosity decreases slowly even in the presence of repeated cycles of self-fertilization (Kellogg, 2001). Moreover, Genotyping-by-Sequencing reads were aligned to the *S. bicolor* reference genome (McCormick et al., 2018); alignment of sequences from an allotetraploid to a diploid genome can result in an overestimation of heterozygous loci due to alignment of homeologs. In *S. halepense* homeologs descending from orthologs in the genomes of its diploid ancestors, *S. bicolor* and *S. propinquum*, are maintained. However, following hybridization with *S. bicolor* it is difficult to predict the behavior of such homeologs across generations, given the different possibilities of chromosome pairing at meiosis (Rakshit et al., 2016). It is nonetheless expected that at least some of the homeolog chromosome pairs can be maintained and contribute to increasing the genetic variability of the recombinant inbred lines. Finally, a possible bias in the calculation of allelic frequencies in Sb × Sh can be due to the impossibility of determining allele dosage in tetraploids given the low coverage inherent in GBS strategies; for instance, at a given genomic DNA site, Ref:Alt allele ratios of 3:1 and 1:3 are detected as normal 1:1 heterozygotes in diploids, but such a heterozygous genotype could be wrongly assigned due to the uncertainty associated with variant calling.

The higher frequency of heterozygous SNPs and the possible genotype calling errors due to homeologs or allele dosage in tetraploids might probably reduce the statistical power of GWAS analysis; nonetheless, the same approach was successful in detecting genomic regions controlling anthocyanins, polyphenols, and tannins contents in a subset of the Sb × Sh population used in this study (Habyarimana et al., 2019). In addition, the great majority of significant SNPs found in this study were polymorphic in both populations, indicating that they most likely originated in *S. bicolor* genome. However, a few markers only polymorphic in the Sb × Sh group were also significantly associated to maturity (Chr6_29554814, Chr9_42350413, and Chr10_59784957; Table 2), possibly highlighting genetic loci associated to the short life cycle of the weedy parent *S. halepense*. On the other hand, SNPs polymorphic only in Sb might in theory represent genetic variation associated to sorghum domestication, and therefore absent in the wild *S. bicolor* progenitor of *S. halepense*; however, the limited variability in the *bicolor* subgenome of hybrid Sb × Sh lines is indeed also due to the limited number of parents used in the Sb × Sh hybridizations.

Generally, a *R*^2^ threshold of 15% is adopted to define major effect loci; i.e., polymorphisms explaining at least 15% of the observed phenotype variation (Habyarimana et al., 2019). In our study, however, only one marker reached this threshold (Chr1_8820891, *R*^2^ = 0.16 for DMC), highlighting the strongly quantitative nature of biomass-related traits in which a high number of loci contributing small phenotypic effects are expected rather than a few loci with major effects. Therefore, to search genomic regions for additional candidate genes useful for breeding purposes, we lowered the *R*^2^ threshold to 5%, which led to select a total of 8 markers located on chromosomes 1, 3, 8, and 9 (Table 2). Comparable *R*^2^ values were also considered as relevant for marker-assisted selection in previous studies (Nadeem et al., 2020). A region spanning 500 Kb upstream and downstream each of these markers was analyzed on the sorghum reference genome sequence (Supplementary Table 1). Given the nature of analyzed traits, virtually any gene whose function is related to the plant primary metabolism can be suspected to have an effect, making it very hard to narrow the list of candidates; when Gene Ontology (GO) annotations for “biological process” are considered, for example, the most represented terms in the selected regions are protein phosphorylation (28 genes), regulation of transcription (25 genes), transmembrane transport (16), metabolic process (14) and oxidation-reduction process (12), which can all be theoretically linked to regulation of plant growth and metabolism (Supplementary Table 1). To get the best possible support for the selection of candidates, we decided to focus on the genes closest to the 8 major breeding interests SNPs within the physical interval supported by the LD decay information.

The most important major effect SNP uncovered in this study is Chr1_8820891 that explained 16.2% of the variation for dry mass fraction of fresh material. This marker is in close proximity of transcript Sobic.001G112500 which is a putative zinc-finger homeodomain (ZF-HD) protein (Supplementary Table 1). Interestingly, in a previous GWAS analysis Sobic.001G112500 was associated to midrib color variation (Xia et al., 2018), which is dependent upon the action of the *D* (Dry stalk) locus controlling stalk moisture and juiciness. In addition, midrib color was reported to be highly predictive of sugar yield, albeit not significantly correlated with dry biomass (Burks et al., 2015). The gene determining the *D* locus function is thought to be a NAC transcription factor (Sobic.006G147400, on chromosome 6) whose function is disrupted in homozygous recessive (*dd*) genotypes (Burks et al., 2015); these latter genotypes show an increased expression of a miniature zinc finger (MIF) gene, which in turn might dimerize with ZF-HD transcription factors to suppress their function (Hu et al., 2008). Based on these findings, it can be hypothesized that Sobic.001G112500 encodes a ZF-HD protein acting downstream the *D* locus NAC gene to determine sorghum stem juiciness. Consistently with this hypothesis, marker Chr1_8820891 is placed only 982bp upstream the transcript start site (TSS) of Sobic.001G112500 and explains the highest proportion of phenotypic variation for dry mass content among SNPs detected in our GWAS analysis. Additional studies are needed to determine the expression level of this gene in *D-* and *dd* genotypes, which was not observed in a previous analysis (Burks et al., 2015), but is reported to be high in the stem and internodes in sorghum (Goodstein et al., 2012). Indeed, Sobic.001G112500 can be considered a strong candidate for the modulation of sorghum stalk moisture, juiciness and therefore fresh/dry mass ratio, and the identification of Chr1_8820891 SNP represents one of the major achievements in this work.

A successful use of the Chr1_8820891 marker in marker assisted breeding, has the potential to improve bioenergy conversion efficiency through a better control of the moisture that existed in the biomass at the time of harvesting. Dry mass fraction of the fresh material is an important trait in biomass plant breeding for biofuel production as biomass moisture content can impact both biomass logistics and energy bioconversion. High moisture content of the biomass affects strongly the combustion process such as lowering the flame temperature and/or the boiler efficiency, which can result in several operational problems including incomplete combustion. To overcome these issues, biomass is often dried before combustion, but this strongly influences the economies of the utilization biofuel (Gebgeegziabher et al., 2013). Moisture content of the biomass is also an important logistics parameter: on the one hand, with low moisture levels, transportation energy is mainly used on the useful component of the biomass (dry mass instead of moisture), and, on the other hand, low moisture levels inhibit anaerobic microbial activity, preventing the biodegradation and allowing for safe long-term storage of biomass (Rentizelas, 2016).

In several other cases significant SNPs co-localized with genes whose putative function can be ideally correlated to biomass and maturity traits. Among them, Sobic.009G237900 contains two SNPs with high effects on dry mass yield and encodes a putative plastocyanin; its possible role in photosynthesis might in theory explain its association with DMY, as it is known, for example, that yield can be determined by factors regulating photosynthetic electron transport rate (Ramamoorthy et al., 2018); additional studies are, however, required to investigate this hypothesis. Another SNP with important effect on DMY was Chr8_56708385 which identified a gene (Sobic.008G138100) with no evident connection to biomass, but interestingly it is less than 1.5 kb distant from Sobic.008G138200, the sorghum ortholog of rice *MEL2* (Nonomura et al., 2011; Dhaka et al., 2020). *MEL2* is a RNA-recognition-motif (RRM) protein possessing ankyrin repeats and a RING finger motif involved in germ-cell development and meiosis progression, required for premeiotic G1/S-phase transition (Nonomura et al., 2011); it is not clear, however, whether it is involved in the transition from vegetative to reproductive phase, which could explain its association to maturity and biomass-related traits. Other candidates emerged from our search include genes putatively involved in the regulation of transcription either for nuclear (Sobic.009G250000, Sobic.009G250100) or plastidial genes (Sobic.009G250400, Sobic.009G250700), hormone metabolism (Sobic.009G249900), or plant mineral nutrition (Sobic.009G250300).

In this work, several blocks of highly correlated markers were identified that likely belong to respective common haplotypes displaying SNPs for traits within QTL regions, and SNPs within and flanking putative genes of interest. It is expected that these haplotypes will play important role in marker-assisted selection in sorghum. However, further investigations are necessary to provide corroborating evidences supporting the importance of these genetic factors as the most suitable candidates for modulating the expression of the traits analyzed in this work. Experiments such as KASP (Kompetitive Allele Specific PCR) or qRT-PCR (Real-Time Quantitative Reverse Transcription PCR) are nonetheless required in order to validate the major effects SNPs reported in this work before they are incorporated into breeding technologies such as Marker-Assisted selection.

## Conclusion

Our data suggest that hybridization of domesticated sorghum with *S. halepense* can be useful for enhancing biomass production, especially if negative traits transmitted by the wild parent, such as seed chattering will be eliminated by breeding techniques including crossing and/or backcrossing to *S. bicolor* followed by selection. As an effort toward identifying valuable loci for biomass-related traits in support for breeding programs, we presented a list of significant and major SNP markers uncovered using GWAS analysis based on high quality marker data and 4 years of field trials of phenotypic evaluations. The use high quality marker data and 4-year field trials allowed precise phenotypic data and the power of our statistical analyses. The obtained data provide therefore strong and useful insight into the genetic control of the complex traits evaluated herein. Moreover, we propose the ZF-HD gene Sobic.001G112500 as an interesting candidate for the control of dry/fresh biomass ratio underlying a QTL localized on chromosome 1, whose action is also supported by previous works. Important SNPs and blocks of SNP marker haplotypes were identified and can be used in marker assisted selection for the development of superior sorghum cultivars. Before the major effects SNPs reported in this work are integrated into breeding technologies such as Marker-Assisted selection, they have to be validated through appropriate gene expression experiments such as KASP or qRT-PCR.

## Data Availability Statement

The full sorghum whole-genome GBS SNP genotyping dataset was archived at https://www.ebi.ac.uk/ena/browser/view/PRJEB40970 under the following accessions: Project: PRJEB40970, analyses: ERZ1668234.

## Author Contributions

EH: conceptualization, methodology, investigation, data curation, supervision, project administration, and funding acquisition. EH, PD, and MD: software, writing – original draft preparation, and visualization. EH, PD, MD, SE, and FB: formal analysis and writing – review and editing. All authors have read and agreed to the published version of the manuscript.

## Conflict of Interest

The authors declare that the research was conducted in the absence of any commercial or financial relationships that could be construed as a potential conflict of interest.

## Footnotes

1 https://vcftools.github.io/index.html

2 https://aussorgm.org.au/sorghum-qtl-atlas/
