# Supplementary figures and images for: Genome-Wide Association Study for Biomass Related Traits in a Panel of Sorghum bicolor and S. bicolor × S. halepense Populations
Source: Front Plant Sci. 2020 Nov 12;11:551305. doi: 10.3389/fpls.2020.551305 (PMC7688983; doi:10.3389/fpls.2020.551305)

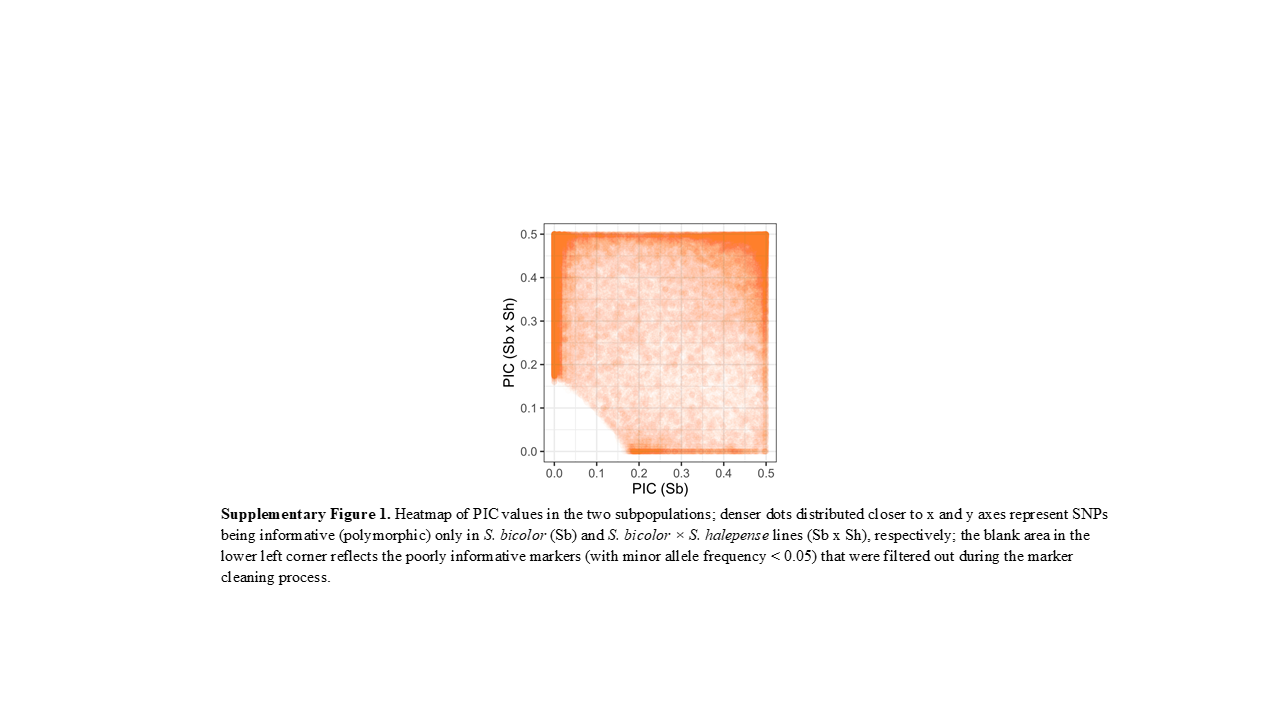

Supplement: Supplementary file 1 [file Image_1.TIF]

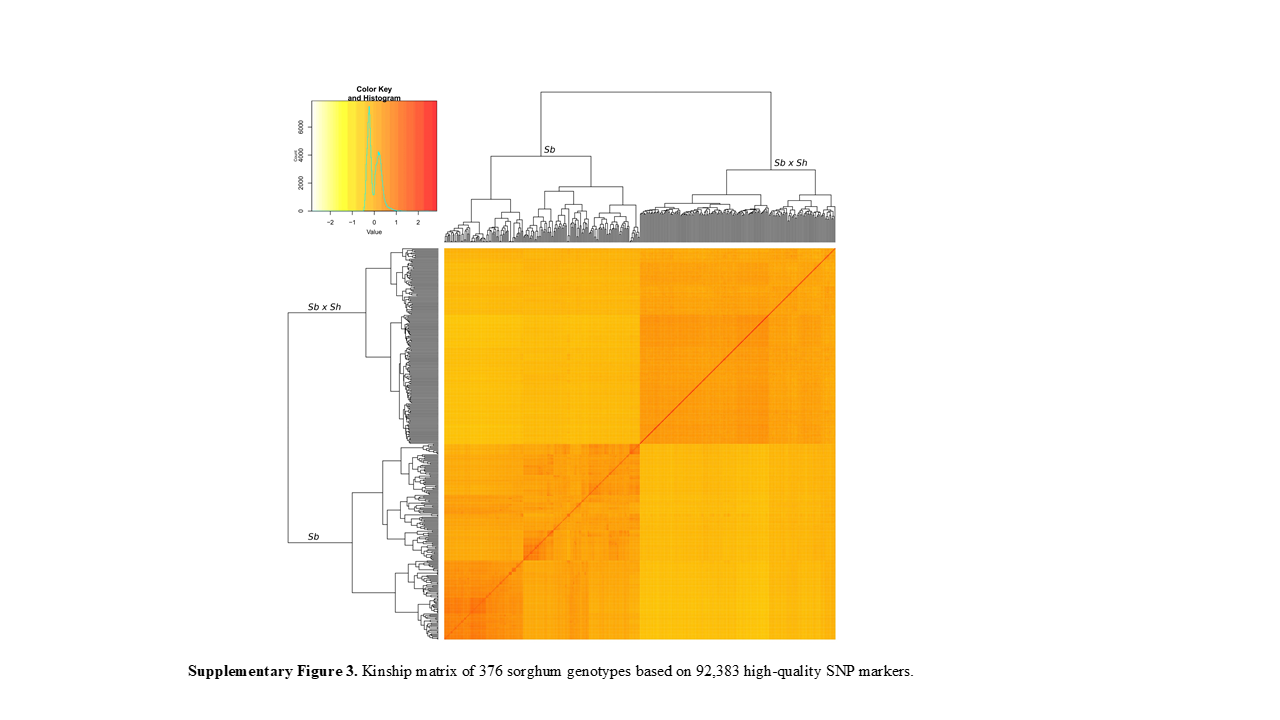

Supplement: Supplementary file 2 [file Image_2.TIF]

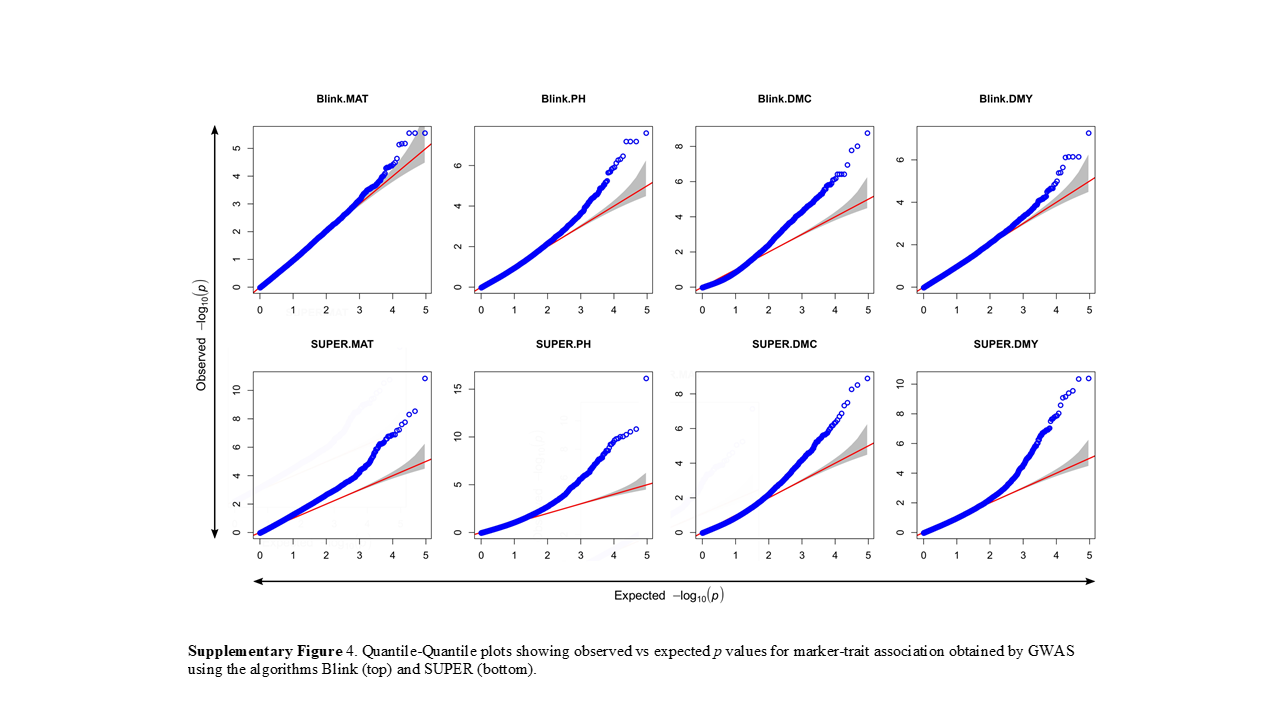

Supplement: Supplementary file 3 [file Image_3.TIF]
